# Frequent House Invasion of *Trypanosoma cruzi*-Infected Triatomines in a Suburban Area of Brazil

**DOI:** 10.1371/journal.pntd.0003678

**Published:** 2015-04-24

**Authors:** Gilmar Ribeiro Jr., Rodrigo Gurgel-Gonçalves, Renato Barbosa Reis, Carlos Gustavo Silva dos Santos, Alekhine Amorim, Sônia Gumes Andrade, Mitermayer G. Reis

**Affiliations:** 1 Laboratório de Patologia e Biologia Molecular, Centro de Pesquisas Gonçalo Moniz, FIOCRUZ-BA, Salvador, Bahia, Brazil; 2 Faculdade Ruy Barbosa DeVry, Salvador, Bahia, Brazil; 3 Laboratório de Parasitologia Médica e Biologia de Vetores, Universidade de Brasília, Brasília, Brazil; 4 Programa de Pós Graduação em Desenvolvimento Regional e Urbano (PPDRU), Unifacs, Universidade Salvador, Salvador, Bahia, Brazil; 5 Laboratório de Entomologia, Laboratório Central de Saúde Pública do Estado da Bahia, Secretaria da Saúde, Salvador, Bahia, Brazil; 6 Laboratório de Doença de Chagas Experimental, Autoimunidade e Imunologia Celular, FIOCRUZ-BA, Salvador, Bahia, Brazil; Universidad de Buenos Aires, ARGENTINA

## Abstract

**Background:**

The demographic transition of populations from rural areas to large urban centers often results in a disordered occupation of forest remnants and increased economic pressure to develop high-income buildings in these areas. Ecological and socioeconomic factors associated with these urban transitions create conditions for the potential transmission of infectious diseases, which was demonstrated for Chagas disease.

**Methodology/Principal Findings:**

We analyzed 930 triatomines, mainly *Triatoma tibiamaculata*, collected in artificial and sylvatic environments (forests near houses) of a suburban area of the city of Salvador, Bahia State, Brazil between 2007 and 2011. Most triatomines were captured at peridomiciles. Adult bugs predominated in all studied environments, and nymphs were scarce inside houses. Molecular analyses of a randomly selected sub-sample (n=212) of triatomines showed *Trypanosoma cruzi* infection rates of 65%, 50% and 56% in intradomestic, peridomestic and sylvatic environments, respectively. We detected the *T*. *cruzi* lineages I and II and mixed infections. We also showed that *T*. *tibiamaculata* fed on blood from birds (50%), marsupials (38%), ruminants (7%) and rodents (5%). The probability of *T*. *cruzi* infection was higher in triatomines that fed on marsupial blood (odds ratio (OR) = 1.95, 95% confidence interval (CI) = 1.22-3.11). Moreover, we observed a protective effect against infection in bugs that fed on bird blood (OR = 0.43, 95% CI = 0.30-0.73).

**Conclusions/Significance:**

The frequent invasion of houses by infected triatomines indicates a potential risk of *T*. *cruzi* transmission to inhabitants in this area. Our results reinforce that continuous epidemiological surveillance should be performed in areas where domestic transmission is controlled but enzootic transmission persists.

## Author Summary

Most triatomines are found in forested environments. However, environmental imbalances can influence the decline of sylvatic vertebrate populations and promote food shortages for triatomines. As a survival strategy, these bugs tend to disperse to houses near forest remnants. We studied the spatial distribution, natural infections by *Trypanosoma cruzi* and feeding patterns of triatomines in a suburban area of Salvador, Bahia State, Brazil between 2007 and 2011. *Triatoma tibiamaculata* was collected in sylvatic, peridomestic and domestic habitats, and a high *T*. *cruzi* infection rate was established for triatomines that fed on blood from birds, marsupials, ruminants and rodents. The frequent invasion of dwellings by infected triatomines can present a risk for oral and vectorial transmission of *T*. *cruzi* to humans. We recommend that residents of triatomine-prone areas perform the following procedures: (i) avoid leaving artificial lights on overnight; (ii) periodically clean palm trees near their homes; and (iii) install net screens on windows. Moreover, education campaigns should promote community awareness of the risks of *T*. *cruzi* infection and provide alternatives to prevent human contact with vectors. Finally, we highlight the importance of continuous epidemiological surveillance in areas where domestic transmission is controlled but enzootic transmission persists.

## Introduction

The demographic transition of populations from rural areas to large urban centers often results in the development of slums, disordered occupation of forest remnants, parks, and protected areas, and increased economic pressure to develop high-income buildings in these same areas. Ecological and socioeconomic factors associated with these urban transitions create conditions for the potential transmission of infectious diseases, which has been demonstrated for Chagas disease [1,2]. Vector-borne *Trypanosoma cruzi* transmission occurs in urban areas of South [2–7], Central [8] and North America [9]. Moreover, several urban outbreaks of acute Chagas disease related to the ingestion of contaminated food were recently described [10–14].

Deforestation, forest fragmentation and human occupation can cause the local extinction of mammals in Atlantic forest remnants [15], thus diminishing food sources for hematophagous insects, such as triatomine bugs, which are vectors of Chagas disease. Furthermore, anthropogenic landscape disturbances can increase triatomine abundance and their rates of infection with *T*. *cruzi*, which indicates that forest remnants may be sources for vector populations within disturbed areas [16,17]. These processes, coupled with socioeconomic factors and triatomine attraction to artificial lights, may influence the invasion of Chagas disease vectors in environments near human populations [18–20].

Recently, the urban growth of Salvador City, Bahia, Brazil [21] has caused a strong and increasing pressure on the remaining forested areas of the town and promoted the conditions required for the invasion of human dwellings by Chagas disease vectors, which have previously been reported in suburban and slums areas in Salvador City [18]. This invasion is concerning because the occurrence of infected triatomines inside homes can contribute to oral outbreaks and vectorial transmission of Chagas disease [22].

The first notification of triatomine bugs in Bahia was provided by Brumpt & Silva [23]. Chagas disease became a serious public health problem in the city of Salvador in the 1950’s, with the registration of numerous cases of autochthonous chagasic cardiopathy, which was associated with vectorial transmission by *Panstrongylus megistus* and *Triatoma rubrofasciata*. Triatomine populations were subsequently controlled with large-scale insecticide campaigns [24]. However, triatomines continue to invade homes in Salvador, which underscores the need to analyze the distribution of *T*. *cruzi* infection among vectors and associated risk factors for Chagas disease re-emergence in this city. This paper studied the spatial distribution, natural infection by *T*. *cruzi* and blood meal sources of triatomines captured between 2007 and 2011 in a suburban area of Salvador City.

## Methods

### Study area

**Fig 1:**
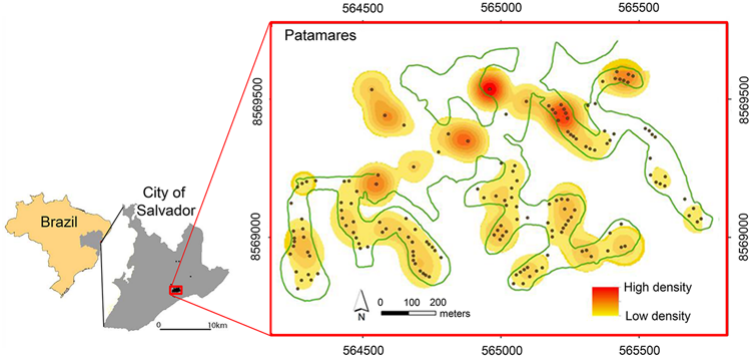
Location of the studied area, Salvador, Brazil. The circles show where triatomines were collected from July 2007 to December 2011. The rectangle shows the location of the Patamares neighborhood where most of the triatomines were captured. Green lines represent forest margin in the area. Kernel density for *Triatoma tibiamaculata* is presented by yellow-red scale (yellow-low; red-high).

This research was conducted in Salvador City from July 2007 to December 2011. The city of Salvador is the capital of Bahia state, which is in the northeast of Brazil (Fig 1). This city was founded within the Atlantic Forest, which is of the richest and most endangered ecoregions in Brazil. Salvador City has an annual average temperature of approximately 25°C with small variation. The climate is tropical, hot and humid with no pronounced dry season and classified as Af according to the Koppen-Geiger classification. The suburban area where triatomines were collected was developed recently near Atlantic Forest remnants. The houses are located in an upscale area where mansions are interspersed with vegetation. Most peridomiciles have swimming pools, gardens, kennels and a barbecue area. Chicken coops, pigsties and corrals were not observed.

### Data collection

Part of the examined triatomines were captured by community households or health workers from the Zoonosis Control Center (ZCC) of Salvador following the recommendations of the Brazilian Ministry of Health [25,26]. Briefly, manual triatomine searches in the houses notified by ZCC were conducted by trained individuals equipped with gloves, flashlights and tweezers. Triatomines were categorized as intradomestic, peridomestic or sylvatic based on the environment where they were captured. Peridomestic triatomines were captured in the area surrounding homes, usually in a fenced compound. Sylvatic triatomines were captured by our field group and ZCC workers by manual searches in *Attalea* spp. palm trees [27]. We registered information for each sample using a standardized entomological survey form. The geographical coordinates of the positive houses were determined by using a handheld GPS. The triatomines were separated by sex and nymphal stage and morphologically identified using taxonomic keys [28]. We performed abdominal dissection and isolation of intestinal contents, which were eluted in phosphate buffered saline (PBS) and subsequently frozen at -70°C until analysis.

### Molecular techniques

DNA extraction was performed using the DNAzol commercial kit (Invitrogen, California, USA). *Trypanosoma cruzi* identification and lineage typing followed previously described PCR protocols [29]. We used specific primers from previous publications [30,31] and new primers developed in this study to evaluate triatomine blood meals (Table 1). The list of animals investigated as probable food sources for triatomines are shown in Table 1. The following PCR conditions were employed: initial denaturation at 95°C for 5 min; 35 cycles of 95°C for 1 min, 57–65°C (see Table 1) for 1 min, and 72°C for 1 min; and final elongation at 72°C for 10 min.

**Table 1:** List of animals investigated as food sources of triatomines, primer sequences, expected size of PCR products, National Center for Biotechnology Information (NCBI) access number, references (Ref.) and PCR annealing temperatures (°C).

Taxa	Primer foward (5'- 3')	Primer reverse (5'- 3')	Size (bp)	Access n°	Ref.	°C
Avian	ATAGAATGGCCTGGGTTGAAAAG	AAGTTTTTCACACAGAGGGTGGT	197	AC092403.4	1	60
*Bos taurus*	TTTTATCCTTCCATTTATCATCATAGC	AAGAGGAAAGAATGGACTGTTTTAGAT	1261	DQ124400.1	1	65
*Canis familiaris*	GTCAATGGTTTCAGGACATATAGTTTT	TATTGTATGCACTTAGTCCTGTTTTTG	476	AY729880.1	2	58
*Homo sapiens*	GTAGTACATAAAAACCCAATCCACATC	GTCGGATACAGTTCACTTTAGCTACC	346	AF381986.1	2	58
*Homo sapiens*	CACCCTATTAACCACTCACG	TGAGATTAGTAGTATGGGAG	470	EU095217	2	57
*Didelphis marsupialis*	ACAGGATCAGAAACCTTTATCTGACTA	TTTGACTTAAAATTTATGGTTTGGTTT	253	Z29573.1	2	59
*Rattus norvegicus*	TATCTGTGTTATTAGACATGCACCATT	TAAAGAGGGTTTAGTTAGGAGGAAAGA	682	X14848.1	2	58
Rodent	CAAGACGGATGATCAAAATGTG	ATTGGGTGGCTGTATATGTATGG	161	AC087102	1	57

1 Walker (2004).

2 This paper.

The PCR products of blood meal samples were analyzed using electrophoresis on 1.5% agarose gels, and *T*. *cruzi* PCR products were separated in 3% agarose gels. The gels were stained with ethidium bromide, visualized under ultraviolet light and photographed in a photo documenter MultiDoc-It (UVP Imaging Systems, USA). A reference size standard (staircase) of 100-bp DNA (Invitrogen, California, USA) was used.

### Data analysis

Triatomine distribution in the studied area was analyzed with a 100-m bandwidth Kernel density estimator and the spatial analyst tool from ArcGis 10.1 (Esri, California, USA). The graphical representation was performed using orthophotos at a scale of 1:2000. Moreover, we estimated the association between triatomine blood sources and *T*. *cruzi* infection using odds ratios (ORs) and 95% confidence intervals (CIs). Descriptive analyses, ORs, 95% CIs and linear regression analyses were performed using the statistical package Epi Info 2000 (CDC, Georgia, USA).

## Results

A total of 167 households (37%) reported the presence of triatomines in the study area, mainly in the Patamares neighborhood, between 2007 and 2011. Increased triatomine notifications coincided with higher temperatures and were frequent between October and March. We obtained 930 triatomines, and most were identified as *Triatoma tibiamaculata* (99%) in the Patamares neighborhood (95%), where a wide distribution of triatomines near forest remnants was observed (Fig 1). The six adult specimens of *Panstrongylus geniculatus* were captured at peridomiciles. Therefore, we have only showed the analyses for *T*. *tibiamaculata*.

**Fig 2:**
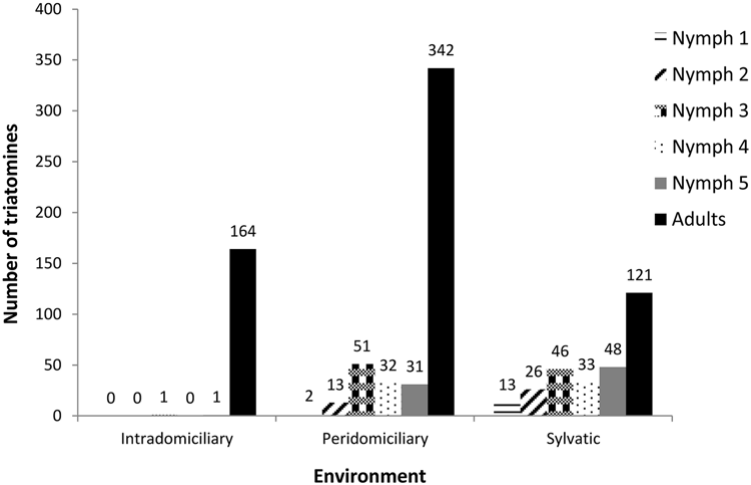
Number of *Triatoma tibiamaculata* collected at different environments from July 2007 to December 2011 in a suburban area of Salvador, Bahia State, Brazil.

Most of the triatomines (n = 471, 51%) were captured in peridomiciles, such as balconies, outdoor kitchens and service areas. Adult bugs predominated in all studied environments, and nymphs were scarce inside houses. However, all triatomine stages were observed in peridomestic and sylvatic environments (Fig 2).

**Fig 3:**
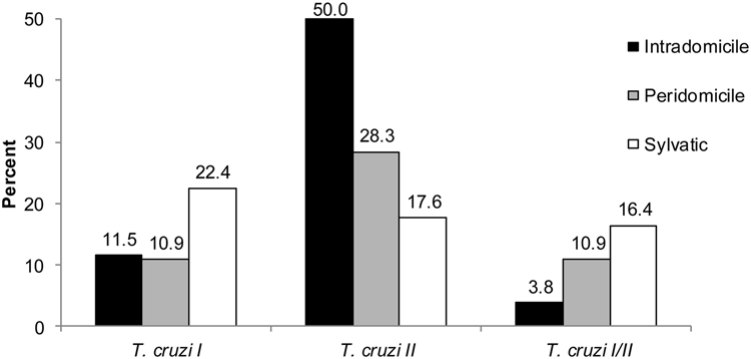
*Trypanosoma cruzi* prevalence (*T*. *cruzi* I, *T*. *cruzi* II and mixed infections) in *Triatoma tibiamaculata* collected in different environments (intradomicile: n = 72, peridomicile: n = 104, sylvatic: n = 36) in a suburban area of Salvador, Bahia State, Brazil.

**Fig 4:**
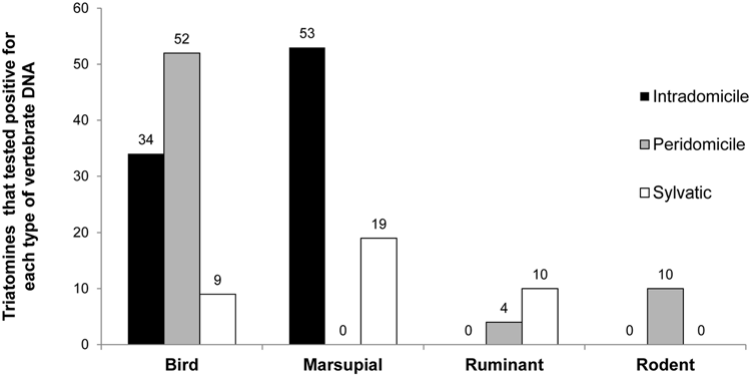
Number of *Triatoma tibiamaculata* that tested positive for each type of vertebrate DNA in different environments in a suburban area of Salvador, Bahia State, Brazil.

**Fig 5:**
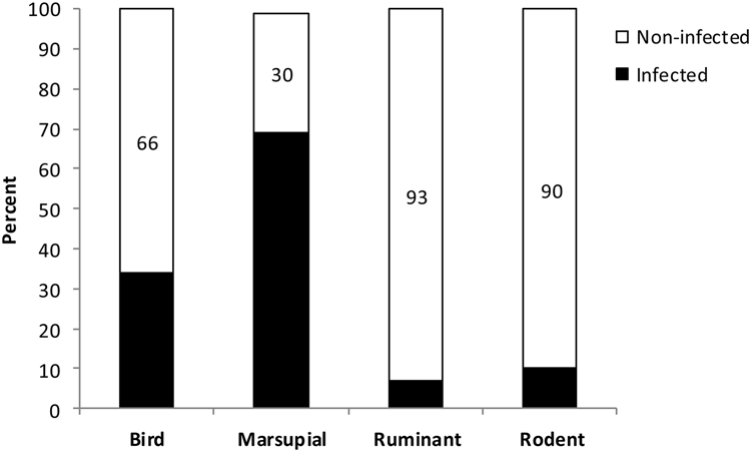
Percentage of each type of blood meal found in *Triatoma tibiamaculata* infected and non-infected with *Trypanosoma cruzi* in a suburban area of Salvador, Bahia State, Brazil.

Molecular analyses of a randomly selected sub-sample (n = 212) of triatomines showed *T*. *cruzi* infection rates of 65%, 50% and 56% in intradomestic, peridomestic and sylvatic environments, respectively. We detected the *T*. *cruzi* lineages I and II and mixed infections (Fig 3). Four blood types were detected in *T*. *tibiamaculata*, and bird and marsupial DNA were the most frequent (Fig 4). Mixed blood meals were detected in 12% of the analyzed samples (bird+marsupial, bird+ruminant, rodent+ruminant). A higher frequency of *T*. *cruzi* infection was observed in triatomine samples with marsupial DNA (Fig 5), and the probability of *T*. *cruzi* infection was higher in triatomines that fed on marsupial blood (OR = 1.95, 95% CI = 1.22–3.11). Moreover, a protective effect against infection was observed in triatomines that fed on bird blood (OR = 0.43, 95% CI = 0.30–0.73).

## Discussion

In the present study we analyzed the spatial distribution, natural infections by *Trypanosoma cruzi* and feeding patterns of triatomines in a suburban area of Salvador, Bahia State, Brazil. *Triatoma tibiamaculata* was collected in sylvatic, peridomestic and domestic habitats, and a high *T*. *cruzi* infection rate was established for triatomines that fed on blood from birds, marsupials, ruminants and rodents. The frequent occurrence of infected *T*. *tibiamaculata* specimens near and inside residences exposes the local population to the potential risk of oral and vector-borne transmission of Chagas disease in suburban areas of Salvador City. The high infection rates and observed association between *T*. *tibiamaculata* and marsupials indicate an intense enzootic transmission of *T*. *cruzi* in Atlantic Forest remnants near houses.

Despite the presence of triatomines in other locations around Salvador City, most of the collection sites were in the Patamares neighborhood. This is a moderate disturbed area, where the houses are interspersed with Atlantic Forest remnants. In this scenario human populations co-occur with triatomines which inhabit sylvatic ecotopes and are benefited by the presence of synanthropic mammals. Moreover, in urban areas with high anthropogenic disturbance (where most houses of Salvador City are located) the number of invasive triatomines should be low due to habitat loss and consequent local extinction of triatomines and sylvatic vertebrate hosts [15,16,17].

High *T*. *cruzi* infection rates (>50%) of triatomines in urban areas were also observed in Chile [31] and Bolivia [6]. The high prevalence of *T*. *cruzi* in *T*. *tibiamaculata* collected from houses in the present study may be caused by their association with marsupials, mainly *Didelphis* spp. These mammals show a synanthropic behavior that facilitates the connection between wild and peridomestic cycles of *T*. *cruzi* [32,33].

Molecular typing demonstrated the presence of *T*. *cruzi* lineages I and II circulating among *T*. *tibiamaculata* specimens. The same lineages were detected in *T*. *tibiamaculata* populations from a Chagas disease outbreak that occurred through oral transmission in southern Brazil [34], and the infection of *T*. *tibiamaculata* with *T*. *cruzi* and its association with marsupials has been previously demonstrated [35, 36].

*Triatoma tibiamaculata* is a species that is primarily associated with palm trees and marsupial and rodent nests in bromeliads [27,35,37]; however, specimens are also found in domestic environments in certain areas of Brazil [38]. The distribution of houses near forest remnants may facilitate contact with infected *T*. *tibiamaculata*. Gottdenker et al. [16,17] observed that anthropogenic disturbances can increase triatomine abundance and infection with *T*. *cruzi* in forest remnants. Consequently, triatomine bugs invade dwellings in search of blood meals or because of their attraction to artificial light sources, which exposes the human population to Chagas disease transmission [18,20].

Birds and marsupials were the most frequent blood meals of *T*. *tibiamaculata* in the studied area. Notably, 10% of the samples did not present an amplification in any of the tested animals, which indicates that primers should be developed with new targets such as bats, which may be important reservoirs for *T*. *cruzi*. Moreover, the specific identification of blood sources using quantitative PCR [39], and sequencing [40] could improve the detection method accuracy and the detection of mixed blood meals.

Most of triatomine data obtained here were from a convenience sample (positive houses reported by the local health service). The bias associated with a non-standardized sampling method is a limitation of the present study and could underestimate the actual occurrence of triatomines. Repeated surveys [41] and improvement of vector surveillance with community participation [42] may increase the detection sensitivity in the house environment and also allow the study of colonization tendencies, considering the finding of nymphs of *T*. *tibiamaculata* inside houses of Patamares neighborhood. Moreover, the spatial distribution of *T*. *tibiamaculata* in forest remnants and houses can be improved from a standard sample in different urban scenarios allowing testing the hypothesis that the occurrence of infected triatomines is dependent on the house distance to forest remnants.

It is important to note that we did not quantify the risk for Chagas disease transmission for the inhabitants of this area and no acute cases of Chagas disease was reported in Salvador city during the last years. In addition, evidence of human blood in the gut of the triatomines was not presented, a serological survey in the human population was not performed, and infection in potential mammal reservoirs was not evaluated. These studies may reveal the actual risk of exposure to *T*. *cruzi* for the inhabitants of this area.

In conclusion, the results show the frequent invasion of infected triatomines in houses, which suggests the potential risk of *T*. *cruzi* transmission to inhabitants in this area. Our results reinforce the need for continuous epidemiological surveillance in areas where domestic transmission is controlled but enzootic transmission persists. We recommend that residents (i) avoid leaving artificial lights on overnight, (ii) perform periodic cleaning of palm trees near their homes, and (iii) install net screens on windows. Moreover, education campaigns should promote community awareness of the risk of *T*. *cruzi* infection and provide alternatives to prevent human contact with vectors.

## Supporting Information

S1 Spreadsheet 01 (Georef): Neighborhood localization and entomological data of 930 triatomines captured between 2007 and 2011. 02 (*T*. *tibiamaculata*): *Trypanosoma cruzi* infection and blood meals of 212 selected *Triatoma tibiamaculata* samples. Legend: 0 = negative, 1 = positive.
(XLSX)

S1 Checklist STROBE Checklist. (DOC)
