# Supplementary material for: Frequent House Invasion of Trypanosoma cruzi-Infected Triatomines in a Suburban Area of Brazil
Source: PLoS Negl Trop Dis. 2015 Apr 24;9(4):e0003678. doi: 10.1371/journal.pntd.0003678 (PMC4409385; doi:10.1371/journal.pntd.0003678)
Supplement: S1 Checklist — (DOC) [file pntd.0003678.s002.doc]

PNTD-D-14-01531-R3

STROBE Statement—Checklist of items that should be included in reports of ***cross-sectional studies***

|  | Item No | Recommendation |
| --- | --- | --- |
| **Title and abstract** | 1 | (*a*) Indicate the study’s design with a commonly used term in the title or the abstract  The study uses cross-sectional design, and this is described in the abstract. |
|  |  | (*b*) Provide in the abstract an informative and balanced summary of what was done and what was found  Done |
| Introduction | | |
| Background/rationale | 2 | Explain the scientific background and rationale for the investigation being reported  Done |
| Objectives | 3 | State specific objectives, including any prespecified hypotheses  Done |
| Methods | | |
| Study design | 4 | Present key elements of study design early in the paper Done |
| Setting | 5 | Describe the setting, locations, and relevant dates, including periods of recruitment, exposure, follow-up, and data collection Done |
| Participants | 6 | (*a*) Give the eligibility criteria, and the sources and methods of selection of participants Done |
| Variables | 7 | Clearly define all outcomes, exposures, predictors, potential confounders, and effect modifiers. Give diagnostic criteria, if applicable Done |
| Data sources/ measurement | 8* | For each variable of interest, give sources of data and details of methods of assessment (measurement). Describe comparability of assessment methods if there is more than one group Done. This is provided in the text of the Methods section. |
| Bias | 9 | Describe any efforts to address potential sources of bias Done |
| Study size | 10 | Explain how the study size was arrived at Done at Methods section |
| Quantitative variables | 11 | Explain how quantitative variables were handled in the analyses. If applicable, describe which groupings were chosen and why Done |
| Statistical methods | 12 | (*a*) Describe all statistical methods, including those used to control for confounding Done |
|  |  | (*b*) Describe any methods used to examine subgroups and interactions Done |
|  |  | (*c*) Explain how missing data were addressed Done |
|  |  | (*d*) If applicable, describe analytical methods taking account of sampling strategy Done |
|  |  | (*e*) Describe any sensitivity analyses Done |
| Results | | |
| Participants | 13* | (a) Report numbers of individuals at each stage of study—eg numbers potentially eligible, examined for eligibility, confirmed eligible, included in the study, completing follow-up, and analysed Done |
|  |  | (b) Give reasons for non-participation at each stage Done |
|  |  | (c) Consider use of a flow diagram Done |
| Descriptive data | 14* | (a) Give characteristics of study participants (eg demographic, clinical, social) and information on exposures and potential confounders Done |
|  |  | (b) Indicate number of participants with missing data for each variable of interest Done. – There were no missing data. |
| Outcome data | 15* | Report numbers of outcome events or summary measures Done |
| Main results | 16 | (*a*) Give unadjusted estimates and, if applicable, confounder-adjusted estimates and their precision (eg, 95% confidence interval). Make clear which confounders were adjusted for and why they were included  Done |
|  |  | (*b*) Report category boundaries when continuous variables were categorized Done |
|  |  | (*c*) If relevant, consider translating estimates of relative risk into absolute risk for a meaningful time period Done |
| Other analyses | 17 | Report other analyses done—eg analyses of subgroups and interactions, and sensitivity analyses  Done |
| Discussion | | |
| Key results | 18 | Summarise key results with reference to study objectives  Done |
| Limitations | 19 | Discuss limitations of the study, taking into account sources of potential bias or imprecision. Discuss both direction and magnitude of any potential bias Done |
| Interpretation | 20 | Give a cautious overall interpretation of results considering objectives, limitations, multiplicity of analyses, results from similar studies, and other relevant evidence  Done |
| Generalisability | 21 | Discuss the generalisability (external validity) of the study results Done |
| Other information | | |
| Funding | 22 | Give the source of funding and the role of the funders for the present study and, if applicable, for the original study on which the present article is based Done – document attached. |

*Give information separately for exposed and unexposed groups.

**Note:** An Explanation and Elaboration article discusses each checklist item and gives methodological background and published examples of transparent reporting. The STROBE checklist is best used in conjunction with this article (freely available on the Web sites of PLoS Medicine at http://www.plosmedicine.org/, Annals of Internal Medicine at http://www.annals.org/, and Epidemiology at http://www.epidem.com/). Information on the STROBE Initiative is available at www.strobe-statement.org.
